# Kidney function and clinical recommendations of drug dose adjustment in geriatric patients

**DOI:** 10.1186/1471-2318-13-92

**Published:** 2013-09-10

**Authors:** Marlies Karsch-Völk, Elisa Schmid, Stefan Wagenpfeil, Klaus Linde, Uwe Heemann, Antonius Schneider

**Affiliations:** 1 Institute of General Practice, University Hospital Klinikum rechts der Isar, Technische Universität München, Munich, Germany; 2 Institute for Medical Statistics and Epidemiology (IMSE), Technische Universität München, Munich, Germany; 3 Institute for Medical Biometry, Epidemiology and Medical Informatics (IMBEI), Universitätsklinikum des Saarlandes, Homburg/Saar, Germany; 4 Department of Nephrology, University Hospital Klinikum rechts der Isar, Technische Universität München, Munich, Germany

**Keywords:** Estimated kidney function, Aged 80 years and older, Drug dose adjustment, Primary care, Literature references

## Abstract

**Background:**

In elderly patients chronic kidney disease often limits drug prescription. As several equations for quick assessment of kidney function by estimating glomerular filtration rate (eGFR) and several different clinical recommendations for drug dose adjustment in renal failure are published, choosing the correct approach for drug dosage is difficult for the practitioner. The aims of our study were to quantify the agreement between eGFR-equations grouped by creatinine-based or cystatin C-based and within the groups of creatinine and cystatin C-based equations and to investigate whether use of various literature and online references results in different recommendations for drug dose adjustment in renal disease in very elderly primary care patients.

**Methods:**

We included 108 primary care patients aged 80 years and older from 11 family practices into a cross-sectional study. GFR was estimated using two serum creatinine-based equations (Cockroft-Gault, MDRD) and three serum cystatin C-based equations (Grubb, Hoek, Perkins). Concordance between different equations was quantified using intraclass correlation coefficients (ICCs). Essential changes in drug doses or discontinuation of medication were documented and compared in terms of estimated renal function as a consequence of the different eGFR-equations using five references commonly used in the US, Great Britain and Germany.

**Results:**

In general, creatinine-based equations resulted in lower eGFR-estimation and in higher necessity of drug dose adjustment than cystatin C-based equations. Concordance was high between creatinine-based equations alone (ICCs 0.87) and between cystatin C-based equations alone (ICCs 0.90 to 0.96), and moderate between creatinine-based equations and cystatin C-based equations (ICCs 0.54 to 0.76). When comparing the five different references consulted to identify necessary drug dose adjustments we found that the numbers of drugs that necessitate dose adjustment in the case of renal impairment differed considerably. The mean number of recommended changes in drug dosage ranged between 1.9 and 2.5 per patient depending on the chosen literature reference.

**Conclusions:**

Our data suggest that the choice of the literature source might have even greater impact on drug management than the choice of the equation used to estimate GFR alone. Efforts should be deployed to standardize methods for estimating kidney function in geriatric patients and literature recommendations on drug dose adjustment in renal failure.

## Background

Family physicians see elderly multimorbid patients receiving a multiplicity of drugs on a daily basis. In many of these patients chronic kidney disease is complicating drug prescription. Family physicians are faced with the challenge to decide how to best measure renal function and draw conclusions for drug dose adjustment. It is essential that kidney function is assessed correctly and that drug doses are adjusted according to kidney function. Without drug dose adjustment in the case of impaired kidney function resulting in reduced drug clearance, drugs would accumulate and patients would be exposed to the risk of toxicity and adverse drug reactions. Therefore kidney function must not be overestimated. On the other hand, underestimation of kidney function could lead to underdosing or unnecessary discontinuation of potentially essential drugs.

If serum-creatinine is used alone for drug dose adjustment without calculating an estimated glomerular filtration rate (eGFR), patients are exposed to an increased risk of adverse drug reactions, because renal function can be considerably impaired despite normal serum-creatinine [1]. Serum creatinine-based equations for calculating an eGFR are important tools for identifying geriatric patients with chronic kidney disease (CKD) and for allocating appropriate drug dosage in these patients [2]. The most commonly used serum creatinine-based GFR or creatinine clearance prediction equations are the abbreviated (4-variable) Modification of Diet in Renal Disease (MDRD) [3] and the Cockroft Gault (CG) formula [4]. However, several authors point out that CG and MDRD have insufficient precision. MDRD is not suitable for identifying patients with early or moderate stages of disease, and CG tends to underestimate kidney function, especially in the elderly. Therefore these formulas should only be used with caution for drug dose adjustment in the elderly [5-8].

An alternative marker for kidney function is serum cystatin C, which has been widely examined and compared with the diagnostic use of serum creatinine. According to two meta-analyses the diagnostic accuracy of cystatin C for impaired renal function ranges between slightly and clearly better than the diagnostic accuracy of creatinine [9,10]. Especially in elderly patients, cystatin C appears to be a suitable marker for assessing renal function because there is evidence that it is less sensitive to metabolic and extra renal factors than creatinine [11]. There are a large number of cystatin C-based equations for estimating GFR. Several of these have been evaluated in elderly patients in comparison to a reference standard. Hojs et al. [12] showed, in an analysis of 234 patients aged 65 years and older, that serum creatinine-based formulas (i.e. MDRD-4-variable) had slightly lower diagnostic accuracy than cystatin C-based formulas using ^51^CrEDTA clearance as a reference standard. The Hoek-formula [13] had the highest accuracy and the Grubb-formula [14] and the Perkins-formula (Simple Cystatin C formula) [15], showed reasonable accuracy compared to ^51^CrEDTA clearance. A German study examined if there was a sufficient drug dose adjustment according to the results of several eGFR-equations based on cystatin C or creatinine in patients 60 years and older who were treated by an emergency physician. The number of patients without appropriate dose adjustment according to kidney function varied considerably depending on the equation used for calculating eGFR. This was because eGFR values themselves differed notably [16]. Swedish researchers investigated renal function in patients living in nursing homes aged 65 years and older using the CG, MDRD and Grubb equation and identified renal risk drugs. Here the authors found poor concordance between the results of different eGFR equations [17].

For patients with impaired renal function, changes in drug prescription are required. Numerous literature references for drug dose adjustment in renal impairment are commonly used in family practice in different countries. Family practitioners rely on literature that can easily be used in office or at the bedside such as small books or handheld devices [18-22].

The multitude of eGFR-equations based on serum creatinine or serum cystatin C that have been proposed and the variety of literature sources for drug dose adjustment in renal failure have resulted in uncertainty in daily practice about which method of estimating kidney function and which literature source should be used.

The aims of our study were

1) to quantify the agreement between eGFR-equations grouped by creatinine-based or cystatin C-based and within the groups of creatinine and cystatin C-based equations and

2) to investigate whether the use of various literature and online references results in different recommendations for drug dose adjustment in renal disease in very elderly primary care patients.

## Methods

### Patients and patient data

We included primary care patients aged 80 years and older, enlisted to 11 participating family practices in Bavaria, a federal state in southern Germany. We recruited practices by sending a letter of inquiry to the 120 teaching practices associated to the Insitute of General Practice at the Technical University Munich. Eleven practices agreed to participate. The practices were instructed to ask their patients 80 years and older if they would participate in this study. Every practice was supposed to choose at least ten patients. Patients were examined in practice, at home visits in their residence or in their nursing homes. We excluded patients unable to provide full informed consent, with thyroid dysfunction not normalized with treatment, with high dose corticosteroid therapy or with cancer, because serum cystatin C levels are influenced by these factors. As patients had to be able to give written informed consent, only patients without evidence of dementia were included. Blood samples were drawn for measuring cystatin C, creatinine, and thyroid-stimulating hormone (TSH). In addition, body height, weight, prescribed medication and diagnoses (coded according to the International Classification of Diseases (ICD)) were collected as documented in the physicians’ patient chart. All participants gave written informed consent.

### Ethics statement

The study was approved by the Ethical Committee of the Medical Faculty of the Technical University Munich.

### Laboratory measurements

A particle-enhanced immuno-nephelometric assay (Prospec Siemens) was used for measuring cystatin C levels, a colorimetric method for measuring creatinine levels, an enzymatic UV-Test (Urease IGLDH, Roche Cobas 8000) for measuring urea levels. Sodium and potassium levels were determined indirectly by ionselective electrodes (Roche Cobas 8000) and TSH levels by an electrochemical luminescence immunoassay (Roche Cobas e 411). Blood count was analysed using a Sysmex analyser (XE 2100).

### Estimation of glomerular filtration rate

GFR was estimated using two serum creatinine-based equations and three cystatin C-based equations which are listed below:

Cockroft-Gault equation:

$$\begin{array}{ll} {\mathrm{eGFR}}_{\mathrm{ml}/\min/1.73m2}= & \left[\left(140‒\mathrm{age}{\;}_{\mathrm{years}}\right){\times \mathrm{weight}}_{\mathrm{kg}}\right]/\left(72\times \right)\\ & \left({\mathrm{CREA}}_{\mathrm{mg}/\mathrm{dl}}\right)\times 1.73/\mathrm{body}\;\mathrm{surface}\\ & {\mathrm{area}}_{m2} \end{array}$$

[4]

MDRD equation:

$$\begin{array}{ll} {\mathrm{eGFR}}_{\mathrm{ml}/\min/1.73m2}= & 175\times {\left({\mathrm{CREA}}_{\mathrm{mg}/\mathrm{dl}}\right)}^{-1.154}\times \\ & {\left({\mathrm{age}}_{\mathrm{years}}\right)}^{-0.203}\left(\times 0.742\;\mathrm{for}\;\mathrm{women}\right) \end{array}$$

[3]

Grubb equation:

$$\begin{array}{ll} {\mathrm{eGFR}}_{\mathrm{ml}/\min/1.73m2}= & 84.69\times {\mathrm{cystatin}\;C}_{\mathrm{mg}/l}{}^{-1.680}\\ & \left(\times 0.948\;\mathrm{if}\;\mathrm{female}\right) \end{array}$$

[14]

Hoek equation:

$${\mathrm{eGFR}}_{\mathrm{ml}/\min/1.73m2}=-4.32+80.35/{\mathrm{cystatin}\;C}_{\mathrm{mg}/l}$$

[13]

Perkins equation:

$${\mathrm{eGFR}}_{\mathrm{ml}/\min/1.73m2}={100/\mathrm{cystatin}\;C}_{\mathrm{mg}/l}$$

[15]

Serum creatinine-based equations were the CG equation [4] (adjusted for body surface area by the DuBois formula [23]: ${\mathrm{BSA}}_{m2}{=0,007184\times \mathrm{height}}_{\mathrm{cm}}^{0.725}{\times \mathrm{weight}}_{\mathrm{kg}}^{0.425}$) and the abbreviated (4-variable) MDRD [3]. The cystatin C-based eGFR-equations were the Grubb equation [14], the Hoek equation [13], and the Perkins equation (Simple Cystatin C formula) [15]. The CG and MDRD equation were chosen because they are commonly in use and well known to practitioners. Two cystatin C-based equations (Hoek and Grubb) were chosen because they were developed on data from European study populations and therefore seemed suitable for our study population. The Perkins equation was chosen because its use would be practical for daily use also on home visits as it can easily be calculated on a pocket calculator because of its simplicity.

### Verification of drug dose adjustment to renal function

For each eGFR-value, each patient’s drug doses of his or her actually prescribed medication according to estimated renal function were verified using several literature references. In our study, five different references, mentioned in the Background section were consulted. We chose references that are easily accessible and commonly used in daily clinical practice: (e.g. in the U.S. [18], in Great Britain [19,20] and in Germany [21,22]). More extensive pharmaceutical literature references were not included as we aimed to examine tools that are suitable for daily use in medical practice. Essential changes in drug doses or discontinuation of medication were documented and compared in terms of estimated renal function as a consequence of the different eGFR-equations.

Each participating family practice received a detailed report about every single patient including results of laboratory tests, estimated kidney function according to different equations and recommendations on adjustment of the prescribed drugs according to eGFR results and literature recommendations. We did not assess if participating family practitioners implemented our recommendations.

### Statistical analysis

All statistical analyses were performed using SPSS statistical software (SPSS 18.0 and 19.0, IBM, Somers, NY). Patient data was summarized descriptively (percentage, mean, median standard deviations and minimum/maximum). We created scatter plots to graphically examine concordance between the different eGFR estimations, including a linear trend regression as well as a bisecting line. Concordance was further analysed by calculating intra-class correlation coefficients (ICCs, two-way mixed model) along with a respective 95% confidence interval. Comparisons concerning the numbers of drugs that may require dose adjustments were done using the two-sided Friedman- or Wilcoxon-test, as appropriate. Agreement beyond chance between different literature and online sources regarding the necessity of drug dose adjustment was quantified with kappa statistic. Kappa values < 0.4 were considered as low agreement, values between 0.4 to 0.59 as moderate, 0.6 to 0.74 as good and higher as very good agreement.

## Results

A total of 108 patients, 73% female with a mean age of 85 years, were included in the study. The number of patients enlisted from each individual practice ranged from one to 25 patients. On average five patients per practice were included. Patient characteristics, frequent diagnoses and drugs, and laboratory measures are summarized in Tables 1, 2 and 3. Chronic kidney failure was documented a priori for 32 patients (30%) in the general practitioners´ patient records. It was not possible to determine if drug dose adjustment was done before by the general practitioners in that group due to kidney function or due to other reasons. The three most frequent diagnoses (prevalence over 50%) were hypertension, arthrosis and cardiac failure. Due to exclusion of patients unable to provide written informed consent, the proportion of patients suffering from dementia and the proportion of patients taking psychotropic substances were comparatively low. Most commonly taken remedies (taken by more than 50% of the patients) were angiotensin-converting enzyme inhibitors, hydrochlorothiazide and beta-adrenoceptor blockers. Some of these drugs may require drug dose adjustment in renal impairment as shown in Table 3.

**Table 1:** Physical and biochemical patient characteristics

**N = 108**	**N (%) or Median (range)**
Female sex	79 (73)
Age (years)	85 (80–102)
Body Mass Index (kg/m^2^)	26.0 (16.7-47.1)
Number of diagnoses	9 (2–17)
Number of drugs/day	7 (1–19)
Creatinine (mg/dl)	1.0 (0.5-3.2)
Cystatin C (mg/l)	1.2 (0.6-3.4)
eGFR Cockroft-Gault (ml/min/1,73 m^2^)	45.3 (12.2-95.8)
eGFR MDRD (ml/min/1,73 m^2^)	53.2 (14.8-118.1)
eGFR Grubb (ml/min/1,73 m^2^)	63.5 (11.1-174.5)
eGFR Hoek (ml/min/1,73 m^2^)	65.5 (19.7-123.2)
eGFR Perkins (ml/min/1,73 m^2^)	87.0 (29.9-158.7)

**Table 2:** Most common diagnoses as documented in patient charts as coded in ICD 10

**N = 108**	**N (%)**
Hypertension	94 (87)
Arthrosis	58 (54)
Cardiac failure	54 (50)
Hyperlipidemia	49 (45)
Type 2 diabetes mellitus	43 (40)
Coronary heart disease	35 (32)
Chronic kidney failure (no dialysis required)	32 (30)
Hyperuricemia	29 (27)
Atrial fibrillation	28 (26)
Depression	21 (19)
Incontinence	21 (19)
Peripheral artery occlusive disease	21 (19)
Struma	19 (18)
Dementia	13 (12)
Chronic obstructive pulmonal disease (COPD)	12 (11)

**Table 3:** Most commonly taken remedies

**N = 108**	**N (%)**
Angiotensin-converting enzyme inhibitor*	59 (55)
Hydrochlorothiazide*	59 (55)
Beta-adrenoceptor blocker**	54 (50)
Acetylsalicylic acid*	41 (38)
Non steroidal anti inflammatory drugs*	23 (21)
Statins**	22 (20)
Antidepressants**	18 (17)
Opioids**	18 (17)
Sartans**	17 (16)
Allopurinol*	10 ( 9)
Bisphosphonates*	8 ( 7)
Neuroleptics**	8 ( 7)
Sulfonylureas*	8 ( 7)
Acetaminophen (Paracetamol)*	7 (.7)
Metformin*	7 ( 7)
Metoclopramide*	7 ( 7)
Aldosterone antagonists*	4 ( 4)
Antidementives**	2 ( 2)
Antiepileptics**	2 ( 2)
Alpha2 adrenergic receptor antagonists*	2 ( 2)
Digoxin*	2 ( 2)
H2 receptor antagonists*	2 ( 2)
Disease modifying antirheumatic drugs**	2 ( 2)

* All drugs in this class require dose adjustment in case of moderate or severe renal impairment.

** Some drugs of this class require dose adjustment in case of renal impairment.

**Figure 1:**
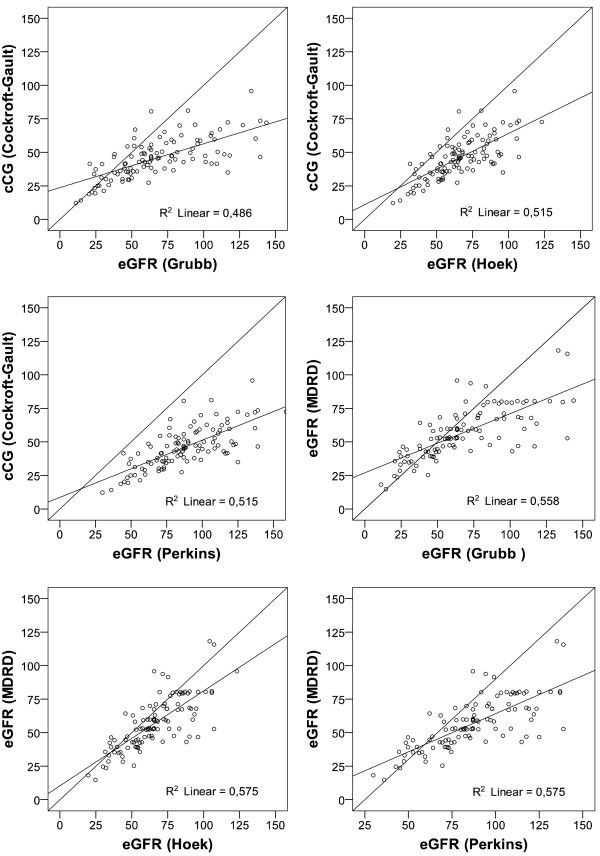
**Scatter plots illustrating the relation between the results of the two creatinine-based and the three cystatin C-based equations.***Figure Legend:* R^2^ Linear: linear regression coefficient. CG = Cockroft Gault; MDRD = Modification of Diet in Renal Disease.

The lowest median estimate of renal function resulted from the use of the creatinine-based CG and MDRD equations and the highest from the cystatin C-based Perkins-equation (Table 1, lower section). High concordance was observed between the creatinine-based equations alone and between the cystatin C-based equations alone (Table 4). Concordance between creatinine-based equations and cystatin C-based equations was moderate. Scatterplots illustrating the relation between the results of the two creatinine-based and the three cystatin C-based equations are presented in Figure 1. Concordance was highest between the creatinine-based equations and the Hoek equation, and lowest between the creatinine-based equations and the Grubb equation.

**Table 4:** Intraclass-correlation coefficients for eGFR according to different equations

**eGFR (ml/min/1.73 m**^**2**^**)**	**CG (95% CI)**	**Hoek (95% CI)**	**Grubb (95% CI)**	**Perkins (95% CI)**
MDRD	0.87 (0.81-0.91)	0.76 (0.66-0.83)	0.65 (0.53-0.75)	0.73 (0.63-0.81)
CG		0.69 (0.57-0.78)	0.54 (0.39-0.66)	0.63 (0.50-0.73)
Hoek			0.90 (0.85-0.93)	0.98 (0.97-0.98)
Grubb				0.96 (0.95-0.98)

CG = Cockroft-Gault; MDRD = Modification of Diet in Renal Disease;

95% CI = 95% confidence interval.

When comparing the five different references consulted to identify necessary drug dose adjustment in relation to the estimated kidney function, we found that the number of drugs that necessitate a dose adjustment in case of renal impairment differ considerably (Table 5). “Drug Prescribing in Renal Failure” and “Arzneimittel Pocket” identify the highest number of drugs which need to be adjusted following renal impairment. None of the literature references contained information on all drugs taken by the study patients.

**Table 5:** Differences in the number of drugs that may require dose adjustment in kidney failure per patient according to the five literature sources

**Nr. of Drugs**	**Renal Drug HB**	**Dosing**	**AMP**	**BNF**	**DPRF**
0	7 (6%)	4 (13%)	9 (8%)	11 (10%)	7 (7%)
1	37 (34%)	19 (18%)	18 (17%)	23 (21%)	16 (15%)
2	33 (31%)	40 (37%)	28 (26%)	40 (37%)	34 (32%)
3	21 (19%)	25 (23%)	36 (33%)	25 (23%)	31(29%)
4	9 (8%)	7 (6%)	13 (12%)	7 (7%)	12 (11%)
5	1 (1%)	3 (3%)	3 (3%)	1 (1%)	5 (5%)
6			1 (1%)	0 (0%)	3 (3%)
7				1 (1%)	
M (SD)	1.92 (1.10)	2.01 (1.20)	2.36 (1.26)	2.02 (1.20)	2.48 (1.34)

Friedman-test for comparison of all literature sources: p < 0.001; Wilcoxon-test paired samples: p < 0.001 for Renal Drug HB vs. AMP, Dosing vs. AMP, Renal Drug HB vs. DPRF, Dosing vs.DPRF, AMP vs. BNF, BNF vs. DPRF, and p = 0.39 for Renal Drug HB vs. Dosing, p = 0.36 for RDB vs. BNF, p = 0.95 for Dosing vs. BNF, p = 0.22 for AMP vs. DPRF;

M = Mean, SD = Standard deviation;

Renal Drug HB = The Renal Drug Handbook (19), Dosing = http://www.dosing.de (21), AMP = Arzneimittel Pocket (22), BNF = British National Formulary (20), DPRF = Drug Prescibing in Renal Failure (18).

Agreement and disagreement in recommendations regarding the number of medication changes according to the different literature and online references is exemplarily shown for the results of the CG equation in Table 6. If a literature reference did not provide information about drug dose adjustment for a certain drug, we rated it as “no change necessary”. This approach was chosen because we assumed that a practitioner would not adjust the drug dose in that case. Kappa values ranged between 0.10 and 0.62 with the majority of values below 0.40 (indicating low agreement beyond chance).Testing Kappa coefficients with regard to other eGFR estimations yielded similar results. Descriptive analysis also shows differences in recommendations according to literature references (not shown in table). For example, according to “The Renal Drug Handbook” a change of drug prescription is recommended in 6 to 12 patients depending on the formula used, while according to “Drug Prescribing in Renal Failure” the number of patients varies between 21 and 27. In addition, partially conflicting recommendations were found.

**Table 6:** Agreement beyond chance (Kappa coefficients) among different references regarding necessary drug changes when using the Cockroft-Gault equation for estimating eGFR

	**Dosing**	**AMP**	**BNF**	**DPRF**
	**(95% CI)**	**(95% CI)**	**(95% CI)**	**(95% CI)**
Renal Drug HB	0.15	0.10	0.23	0.20
	(0.00-0.32)	(0.00-0.34)	(0.06-0.40)	(0.03-0.36)
Dosing		0.62	0.51	0.19
		(0.47-0.78)	(0.35-0.68)	(0.02-0.35)
AMP			0.49	0.19
			(0.34-0.65)	(0.05-0.34)
BNF				0.57
				(0.40-0.73)

95% CI = 95% confidence interval;

Renal Drug HB = The Renal Drug Handbook (19), Dosing = http://www.dosing.de (21), AMP = Arzneimittel Pocket (22), BNF = British National Formulary (20), DPRF = Drug Prescibing in Renal Failure (18).

## Discussion

We found that cystatin C-based equations resulted in more optimistic estimations of kidney function than the creatinine-based ones. However, variations in recommendations according to the different literature sources led to more variations in drug dose adjustment than the different GFR-equations alone.

In general creatinine-based equations resulted in a lower eGFR-estimation and in a higher necessity of drug dose adjustment than Cystatin C-based equations. The CG equation resulted in the lowest and the Perkins equation in the highest estimation of kidney-function. Several trials showed that kidney function in elderly patients is estimated to be lower or even underestimated by the CG equation compared to the 4-variable MDRD equation [2,7,24,25]. Results concerning differences in accuracy of CG and MDRD equations in senior patients are inconsistent. Some trials showed higher precision for MDRD than CG when compared with a reference standard [25-27]. Other studies using a reference standard found a lack of precision in both formulas in elderly patients [6,24].

There is little data about the use of cystatin C-based GFR-equations in very old patients. Systematic reviews comparing several studies about diagnostic accuracy of serum cystatin C and serum creatinine for diagnosing renal failure found more studies favouring cystatin C, or describing cystatin C as equal, than those favouring creatinine [28,29]. A systematic review [30] analysed 12 studies comparing serum creatinine, CG equation, MDRD equation, serum cystatin C and several different GFR equations with a reference standard in elderly patients aged 65 years and older. The CG and MDRD equations and the serum cystatin C equations produced the highest correlations with the reference standard. Serum creatinine correlated poorly with the reference standard and therefore appeared to be an insensitive tool for measuring renal function in the elderly.

The impact on drug dose adjustment in four defined drug classes (metformin, nonsteroidal anti-inflammatory drugs, angiotensin-converting-enzyme-inhibitors/angiotensin receptor blockers, and digoxin) depending on different equations was analysed in a Swedish study [17]. As mentioned above, this study also found a lack of concordance between MDRD, CG and the Grubb equation, but the investigated renal risk drugs were rarely prescribed.

In addition to the estimated kidney function, the chosen literature source had a strong impact on the recommendations for drug dose adjustment in our study population. Kappa coefficients reveal a lack of accordance between the recommendations according to the various literature sources. Depending on the literature source, different numbers of drugs are to be adjusted following renal impairment. Also the recommendations concerning critical values of GFR requiring drug dose adjustment differed considerably among the different sources. The use of “Arzneimittel Pocket” resulted in the highest number of advised modifications of medication and the use of “The Renal Drug Handbook” in the lowest. It is unclear why the recommendations are diverging to such an extent. Three of the literature sources (The Renal Drug Handbook, Arzneimittel Pocket and Dosing) have been written by one or more authors and two (British National Formulary and Drug Prescribing in Renal Failure) have been developed by expert panels. All authors point out that they are referring to the newest evidence. The broad variation of results of estimated kidney function and of the recommendations given in different literature and online sources creates an uncertainty for the practitioner. The extent of the risk potential resulting from medication with renal risk drugs for the elderly is unclear.

For daily practice it seems reasonable to use the CG equation as a more conservative approach to dose adjustment in renal impairment. But it is important to keep in mind that there is a risk of underdosing due to the fact that the CG equation tends to underestimate kidney function in the elderly. In borderline cases (e.g. if a drug should be reduced in the case of an eGFR of less than 30 ml/min/1.73 m^2^ and the use of CG is resulting in an eGFR of 29 ml/min/1.73 m^2^) another equation should be used for control. In this case MDRD would work well. In cases where kidney function is over 60 ml/min/1.73 m^2^, a cystatin C-based formula could be used. The use of the Grubb equation makes sense in this case, as it is resulting in a more conservative estimation of kidney function. But we cannot give a clear recommendation, as there are many other creatinine- and cystatin C-based equations that have not been examined in our study.

Regarding the literature recommendations it appears difficult to suggest the use of one particular literature reference since none of the references is covering all of the renal risk drugs. It seems rational to use country related references as they are the most likely to coincide with national guidelines.

A strength of our study is that it was conducted under conditions of routine practice and focused on the consequences for the daily work of a family physician. We examined patients at eighty years of age and older because multimorbidity, polypharmacy and renal impairment are common in this group of patients and, according to literature, it remains unclear which eGFR-estimation is suitable for estimating kidney function. The four literature references and the online-source analysed are easily accessible for practitioners and commonly in use. There are differences in the estimated prevalence of impaired renal function depending on the eGFR-equation applied, and reduced kidney function seems to be common in our study population. A limitation of our study is that kidney function was not determined by inulin-clearance, ^51^Cr-EDTA-clearance, or iohexol-clearance, which are reference standards. However, this was not possible in the primary care setting. But this could not hamper our results related to the heterogeneity of eGFR equations which are originally derived by validation compared to a reference standard. Another limitation is that patients included had to be able to give written informed consent, therefore patients suffering from dementia or severe frailty, could not be examined. As a result, our study population is not fully representative for the age group as the examined patients were in relatively good mental condition. Another limitation of the study may be that we restricted the number of literature references on drug dose adjustment in renal failure to five commonly in use. However, we explicitly wanted to examine practice conditions and examine tools that are easy to apply and can also be used on home visits.

## Conclusions

The chosen literature references resulted in bigger differences in recommendations for drug dose adjustment than the different equations used for estimating eGFR alone. Both, the divergent results of the eGFR equations and the conflicting instructions according to the literature sources can lead to uncertainty for the practitioner and as a result compromise patient safety. Thus there is a strong need for a validated eGFR-equation for estimating kidney function in the elderly. Further research on estimation of GFR and the development of an international consensus on concordant recommendations for practitioners concerning drug dose adjustment in renal failure are desirable.

## Abbreviations

AMP: Arzneimittel pocket [22]; BNF: British National Formulary 20; BSA: Body surface area; CG: Cockroft-Gault; CI: Confidence interval; CKD: Chronic kidney disease; CREA: Creatinine; Dosing: http://www.dosing.de[21]; DPRF: Drug Prescribing in Renal Failure [18]; eGFR: Estimated glomerular filtration rate; GFR: Glomerular filtration rate; ICCs: Intraclass correlation coefficients; M: Mean; MDRD: Modification of Diet in Renal Disease; N: Number of patients; Renal Drug HB: The Renal Drug Handbook [19]; SD: Standard deviation; TSH: Thyroid-stimulating hormone.

## Competing interests

The authors declare that they have no competing interests.

## Authors’ contributions

MKV wrote the study protocol, was the study supervisor and was responsible for technical and material support, participated in data collection, participated in the analysis and interpretation of data, and drafted the manuscript. ES collected data and participated in the analysis and interpretation of data. SW and KL performed the statistical analysis and were involved in the interpretation of data. KL helped to write the manuscript. UH contributed to the interpretation of the data. AS conceived the study, contributed to study protocol, was involved in analysis and interpretation of data and helped to write the manuscript. All authors commented on draft versions and read and approved the final manuscript.

## Pre-publication history

The pre-publication history for this paper can be accessed here:

http://www.biomedcentral.com/1471-2318/13/92/prepub
